# Potential Implications of Acid-Sensing Ion Channels ASIC2 and ASIC4 in Gonadal Differentiation of *Dicentrarchus labrax* Subjected to Water Temperature Increase during Gonadal Development

**DOI:** 10.3390/ani14071024

**Published:** 2024-03-27

**Authors:** Kamel Mhalhel, Rosaria Arena, Maria Rizzo, Giuseppe Piccione, Marialuisa Aragona, Maria Levanti, Francesca Aragona, Francesca Arfuso

**Affiliations:** 1 Department of Veterinary Sciences, University of Messina, Viale Giovanni Palatucci SNC, 98168 Messina, Italy; kamel.mhalhel@unime.it (K.M.); rizzom@unime.it (M.R.); gpiccione@unime.it (G.P.); maria.levanti@unime.it (M.L.); francesca.aragona@unime.it (F.A.); farfuso@unime.it (F.A.); 2 Marine Biochemistry and Ecotoxicology Laboratory, Department of Earth and Sea Science, University of Palermo, Via Barlotta 4, 91100 Trapani, Italy; rosaria.arena@unipa.it

**Keywords:** aquaculture, gonads, seabass, temperature variation, sex differentiation, ion channel, ASIC2, ASIC4, gonads

## Abstract

**Simple Summary:**

The seabass, *Dicentrarchus labrax* (*D. labrax*) is a gonochoristic species that shows clearly sex-linked growth. In fact, females grow faster and reach larger sizes than males. The expression of acid-sensing ion channels (ASICs) 2 and 4 (ASIC2 and ASIC4) in the gonads of *Dicentrarchus labrax* subjected to water temperature increased from 15 °C to 20 °C during the gonadal development. Water temperature during gonadal develepment modified both gonadal differentiation and growth in *Dicentrarchus labrax*. The incidence of intratesticular oocytes highlights intersexual cases, strengthening the possibility that water temperature could modulate sex differentiation in *D. labrax*. The results gathered in the study suggest the possible role of acid-sensing ion channels in gonad differentiation and gamete development in *D. labrax*.

**Abstract:**

In this study, the expression and implication of acid-sensing ion channels 2 and 4 (ASIC2 and ASIC4) in the gonadal sex differentiation of *Dicentrarchus labrax* (*D. labrax*), subjected to increasing water temperatures during gonadal development, were evaluated. Two groups were selected: a control group (CG), in which the average water temperature was maintained at 15 °C and increased to 20 °C in 20 days until weaning; and an experimental group (EG), in which the water temperature was retained at 15 °C for 60 days; thereafter, the temperature was increased daily by 0.5 °C until it reached 20 °C up to the weaning time. Ten fish from the CG and 13 fish from the EG were sampled randomly on the 335th day after hatching (dph). A higher percentage of gonad differentiation in ovaries rather than in testes was observed in the EG compared to the CG (*p* = 0.01). ASIC2 and ASIC4 were detected for the first time in *D. labrax* ovaries by indirect immunofluorescence. Both ASIC2 and ASIC4 were expressed in previtellogenic oocytes of ovaries and in scattered cells within some testes, and were most likely intratesticular previtellogenic oocytes in both the CG and EG groups. The CG group showed a higher expression of ASIC4 than the EG cohort (*p* < 0.05). The results gathered in this study revealed the capacity of water temperature to influence both gonadal differentiation and growth in this gonochoristic fish species, and suggests the possible role of ASIC2 and ASIC4 in gonad differentiation and gamete development in *D. labrax*.

## 1. Introduction

In vertebrates, sex determination can be dictated genetically but is also influenced by environmental factors. In this regard, the most common sex determination is temperature-related, which is generally observed in reptiles. In such cases, the incubation temperature during a critical period of development determines the sex of the offspring. This phenomenon is also observed in teleosts [1,2,3,4,5].

Fish are poikilothermic animals (cold-blooded); they show a wide range of temperature tolerance, and their reproductive processes are known to be sensitive to water temperatures [6,7]. The important role of water temperature in sex determination in fish may be due to temperature fluctuations known to occur during embryonic development in extreme environments. The European seabass (*Dicentrarchus labrax*) is a gonochoristic species that does not display secondary sex morphological characters. Furthermore, it exhibits obvious sex-linked growth: it has been observed that females grow faster and reach a larger size than males [7,8,9,10]. Females of this species are observed to be about 30% heavier than the males from 300–400 days after hatching (dph) until over 1000 dph [9]. Indeed, in aquaculture, size grading is usually a method of classification, and this has shown that the largest fish selected at 86 dph are mainly females [3]. It has been shown, using individual tagging, that females are significantly heavier than males as early as 105 dph (1024-degree days at a water temperature above 10 °C), with a constant difference of 40% from 197 to 289 dph [11]. In European seabass, therefore, sex-specific growth occurs before sexual differentiation of the gonads, as differentiation only begins around 128 dph [3]. No sex chromosomes or ‘genetic sex’ are observed in this species, as sex is determined by environmental temperature and a combination of several genes [12]. It has been previously demonstrated that juvenile and larval seabass, when raised in captivity at temperatures between 19–22 °C instead of the typical temperature of around 14 °C, can develop predominantly as male subjects. The percentage of males can reach 100% (usually about 75%) under these conditions, highlighting the influence of temperature on sex differentiation in this species within aquaculture practices. Among other things, males grow about 35% less than females. It has been suggested that temperature-dependent sex determination is mediated by the inhibition of aromatase mRNA expression [13]. According to another study, the inhibition of aromatase causes a reduction in endogenous 17β-Estradiol (E2) synthesis, resulting in phenotypic males [14]. Additionally, different studies conducted in vertebrates reported the ability of E2 to upregulate the expression of acid-sensing ion channels and to exert a rapid potentiating effect on the functional activity of acid-sensing ion channels (ASICs) [15,16]. The ASICs are members of the epithelial sodium channel/degenerin family (ENaC/degenerin family) of the amiloride-sensitive Na^+^ channels superfamily whose features include high permeability to Na^+^, which could be blocked by amiloride [17]. It has been reported that members of the ASIC gene superfamily might be involved in the differentiation of gametes and in the development of eggs after fertilization [18]. Thus, the temperature sex determinism could be the result of Ca^+^ signaling induced by the temperature-modulated kinetics of ASICs channels. A study investigating the sexually dimorphic behaviors of electric fish showed a sex-related difference in the expression of some ion channel types, including ASICs, suggesting a possible role of these channels in regulating gonadal function in fish or their involvement in neurogenomic mechanisms by which sex differences evolve [19]. It has been suggested that, among the ASICs, the ASIC2 and ASIC4 could have a potential role in promoting the proliferation and differentiation of the granulosa layer in the previtellogenic follicles [20]. It is important to know how sex is differentiated and determined in reared seabass as many male specimens have intratesticular oocytes, which indicate a certain sexual liability. Moreover, it is possible to know the onset of the phenomenon of sexual dimorphism at various post-larval stages in European seabass up to an age at which good identification by inspection and evaluation of the gonads is possible. Through this technique, the different growth patterns between males and females can be identified. Although there is evidence that ASICs contribute to the transduction of mechanical, chemical, and nociceptive information in sensory neurons and other cell types, to date, no information on ASICs expression in the gonads of fish as well as on their involvement in sexual differentiation are available.

In view of the above considerations, the aim of the current study was to investigate the effect of increasing water temperatures during the gonadal development on sex differentiation and growth of *Dicentrarchus labrax*. Moreover, the expression of ASIC2 and ASIC4 in the gonads of the same fish was assessed to evaluate their possible involvement in sex determination.

## 2. Materials and Methods

### 2.1. Animals

A total of 1000 larvae obtained from one population of bloodstock of the same genetic origin were randomly divided into a control group (CG) and an experimental group (EG) and subsequently placed into 8 tanks measuring 7 m^3^ each.

**Figure 1:**
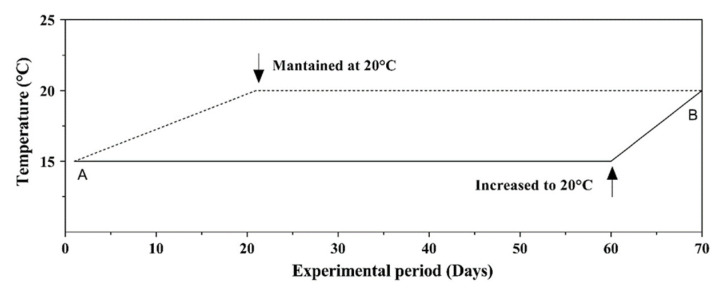
Fast water temperature (°C) increases in control group (CG) expressed with the dotted line and gradual water temperature (°C) increase in experimental groups (EG) expressed with the full line from the start of the study (A) to weaned (B) during the experimental period expressed in days.

In the CG group, the average water temperature was maintained at 15 °C and increased daily by 0.25 °C until it reached 20 °C in 20 days. This temperature condition (20 °C) was kept until the fish were weaned. In the EG tank, the water temperature was maintained at 15 °C for 60 days. Subsequently, the water temperature was increased daily by 0.5 °C until it reached 20 °C, when the seabass specimens were weaned (Figure 1). Other biotic and abiotic parameters of water were monitored (salinity 26–38%; dissolved oxygen 6.5 ppm) and maintained equally in the two groups.

### 2.2. Samples Collection and Histological Analyses

Ten *Dicentrarchus labrax* (*D. labrax*) were collected from CG and 13 *D. labrax* were collected randomly from EG at 335 days post-hatching (dph). Each subject was individually weighed by means of a weighing scale (Kern 440–49 N, Balingen, Germany), and their forks and total lengths were measured using an ictiometer (600 mm; Scubla SNC, Remanzacco, Udine, Italy). The fish were suppressed with a lethal dose of MS-222 (ethil-m-aminobenzoate, 0.4 g/L). A quick break at head level was done with sharp scissors, and the fishes were dissected. Gonads were collected for the evaluation of the degree of gonad differentiation.

### 2.3. Tissue Processing and Histology

The sampled gonads were fixed in Bouin’s solution for 2 h. Subsequently, the samples were dehydrated in a series of graded alcohol solutions, clarified with xylene, and embedded in paraffin. Gonads were then sectioned in 7 µm thick sections and thaw-mounted on microscope slides coated with gelatin before being stained with hematoxylin-eosin. The sexes of individuals were assessed histologically by inspecting the gonad sections.

### 2.4. Immunohistochemistry

The Anti-ASIC4 and ASIC2 (OSR00101W and OSR00098W, Invitrogen-Thermo Fisher Scientific, Rockford, Australia) was declared as being raised against amino acid region 110–160 of human ASIC4 (Uniprot accession number Q96FT7-4) and amino acid region 470–503 of human ASIC2 (Uniprot accession number Q16515-1), respectively. Moreover, datasheets showed that the antibodies were predicted to work with mouse and rat. In order to understand if the used primary antibody, raised against the human ASIC4 and ASIC2 protein, could also work in European seabass, protein alignments were performed for the amino acid region 110–160 of human ASIC4 (Uniprot accession number Q96FT7-4) and amino acid region 470–503 of human ASIC2 (Uniprot accession number Q16515-1) sequence, where the epitope of the Anti-ASIC4 and Anti-ASIC2 antibodies were localized, with Dicentrarchus labrax ASIC4 (A0A8P4G9S7) and ASIC2 (XP_051244303.1). The online software NCBI blastp (protein-protein BLAST: https://blast.ncbi.nlm.nih.gov/Blast.cgi?PAGE=Proteins; accessed on 28 February 2024) was used for peptide alignment.

The alignment of both antibodies’ immunogen sequences and the corresponding protein from the species with reactivity, as mentioned in data sheet, as well as those from the European seabass, were obtained with NCBI blastp (Table 1).

The alignment of the human ASIC4 (Uniprot accession number Q96FT7-4) amino acid region 110–160 and the *D. labrax* ASIC4 (A0A8P4FWP9) showed that the two proteins had 75.51% of amino acids that matched exactly (Identity), and 87% had a similarity, defined either by their chemical properties or based on a point-accepted mutation matrix between the two different sequences, allowing us to hypothesize that the used commercial antibody could work on *D. labrax*. In the same way, the alignment of the human ASIC2 (Uniprot accession number OSR00098W) amino acid region 470–503 and the *D. labrax* ASIC2 (XP_051244300.1) showed that the two proteins had 67.65% of amino acids that matched exactly (Identity), and that 85% had a similarity between the two different sequences, allowing us to hypothesize that the used commercial antibody could work on *D. labrax* (Table 1).

The alignments of both antibodies’ immunogens to the corresponding proteins of mouse and rat showed that 100% identity was achieved, proving the correctness of the immunogen sequence, as the manufacturer reported that the peptides were homologous in rat and mouse.

Parts of the slides were processed for evaluating the expression of ASIC2, and ASIC4 using the immunofluorescence approach. The paraffin slides of the target tissue were deparaffinized in two xylene baths (15 min/each), and rehydrated in 100% ethanol (10 min), 95% ethanol (10 min), 80% ethanol (10 min), 70% ethanol (10 min) and H_2_O (10 min). Deparaffinized slides were rinsed in Tris-HCl buffer (0.05 M, pH 7.5) containing 0.1% bovine serum albumin and 0.1% Triton-X 100. The nonspecific binding was blocked by covering slides with 25% fetal calf serum, after which sections were incubated overnight (at 4 °C) with the primary antibodies: Rabbit polyclonal Anti-ASIC2 Antibody (Invitrogen, Waltham, MA, USA, Cat. # OSR00098W) [1:100], and Rabbit Polyclonal Anti-ASIC4 (Invitrogen, Waltham, MA, USA, Cat. # OSR00101W) [1:100]. After an appropriate rinse, incubation with the following secondary antibodies was performed for 90 min: Goat anti-Rabbit IgG (H + L) Cross-Adsorbed Secondary Antibody, Alexa Fluor™ 594 (Invitrogen, Waltham, MA, USA, Cat. # A-11012) [1:100], and Alexa Fluor™ 594 Donkey anti-Goat IgG (H + L) Cross-Adsorbed Secondary Antibody (Invitrogen, Waltham, MA, USA, Cat. # A-11058). Negative controls were carried out barring the primary antibody, and immuno-labeling was wholly abolished (Supplementary File). Finally, specimen immunolabelling was evaluated by a confocal laser scanning microscope (Zeiss LSM DUO, Carl Zeiss MicroImaging GmbH, Jena, Germany, Europe) with a META module. The image acquisition and evaluation of the fluorescence intensity were made using ZEN 2011 (Black edition) Hist tab (LSM 700 Zeiss software Oberkochen, Germany, Europe).

### 2.5. Statistical Analysis

The number of females and males from the control and experimental groups were compared through Χ^2^ tests. The Kolmogorov–Smirnov normality test was applied on data regarding body weight, total length, and standard length as biometric indices. The resulting data were normally distributed and an unpaired-*t* test was used to determine significant differences in body weight, total length, and standard length between the control and experimental groups. The differences between the arithmetic mean intensity of Asic4 and Asic2 were evaluated using the Mann–Whitney test. Values of *p* < 0.05 were considered statistically significant.

## 3. Results

### 3.1. Histological Results

The histology-based assessment of sex ratios in both the control (CG) and experimental (EG) groups indicated a trend toward a predominantly female population in the EG, accounting for 58.3%. In contrast, the CG group was characterized by a male-dominant population, featuring only 40% females. The discerned disparity in sex ratios between the groups attained statistical significance (Χ^2^ = 6.48; *p* = 0.01) (Table 2).

**Figure 2:**
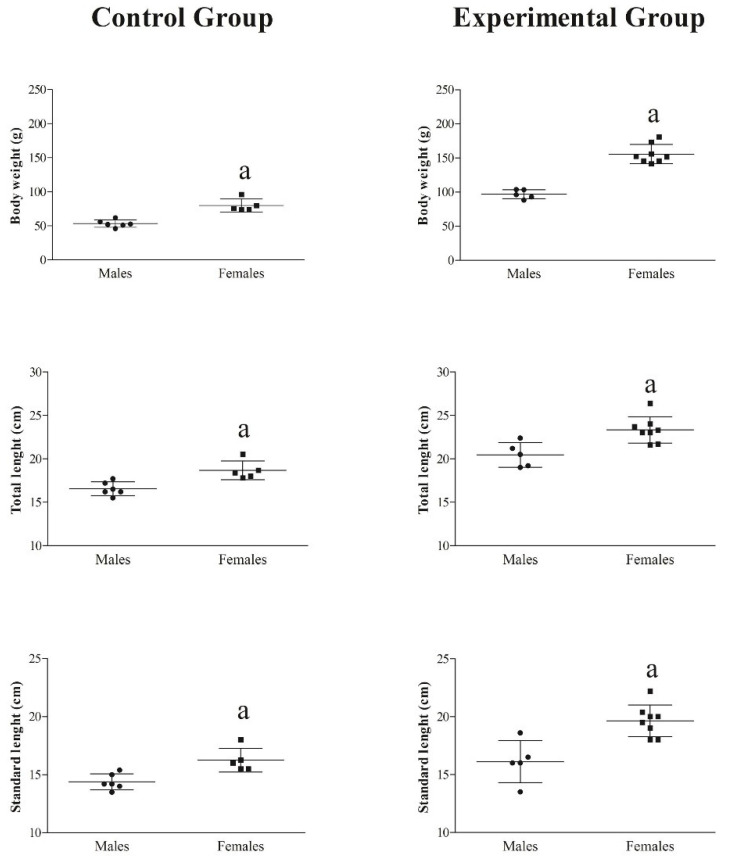
Differences in the body weight, total length, and standard length between males and females of both groups. Significances: ^a^ vs males (*p* < 0.01).

**Figure 3:**
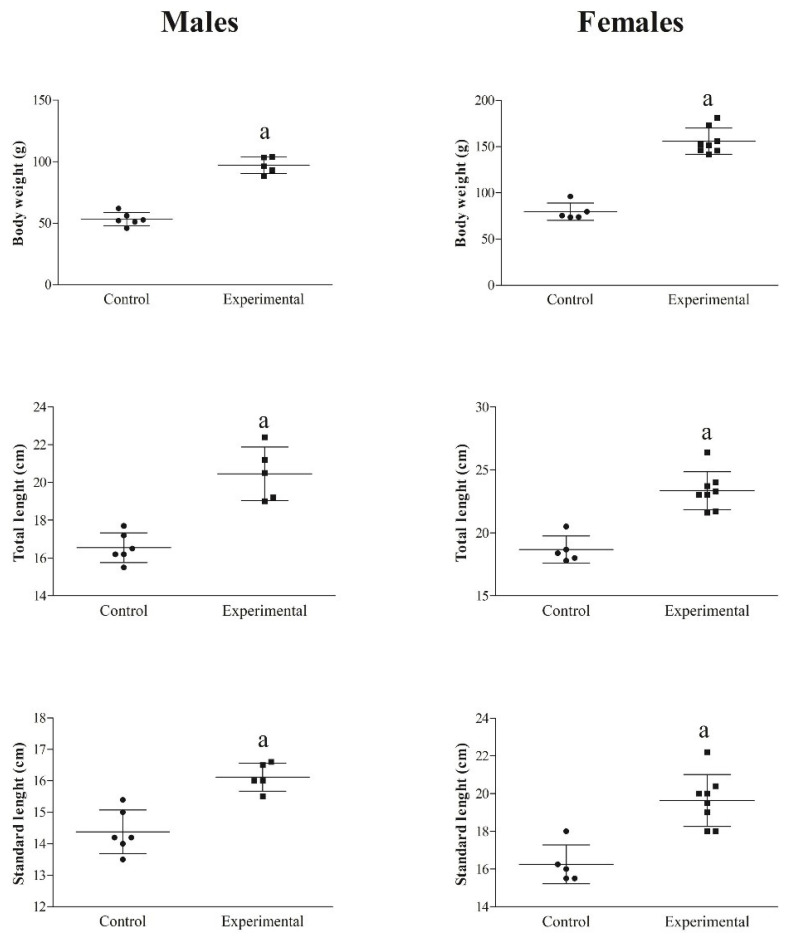
Differences in the biometric indices (body weight, total length, and standard length) between CG and EG. Significances: ^a^ vs control group (*p* < 0.01).

Within each group, all biometric indices were higher in females than males (*p* < 0.01, Figure 2). Body weight, total length, and standard length were statistically significantly lower in CG than in EG (*p* < 0.01, Figure 3).

**Figure 4:**
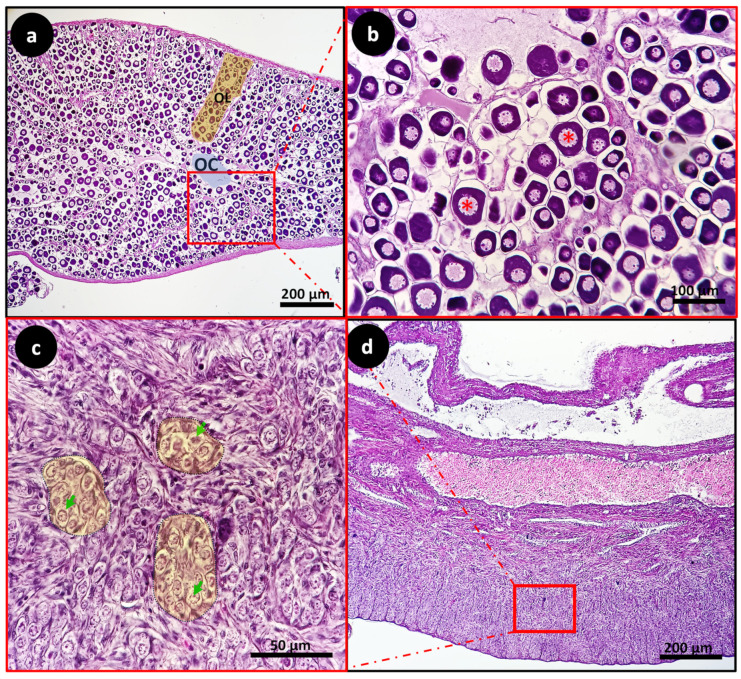
Light micrographs of European seabass gonads from experimental group EG: sagittal-medial section of European seabass ovary (**a**,**b**). Differentiated ovary with well-developed ovarian lamellae (OL) (yellow-shaded), defining the edges of the ovarian cavity (OC) (blue-shaded), full of previtellogenic oocytes (asterisk). Sagittal-medial section of European seabass sexually immature testis (**c**,**d**) with type A spermatogonia (green arrowheads) between the seminiferous lobules (yellow-shaded).

**Figure 5:**
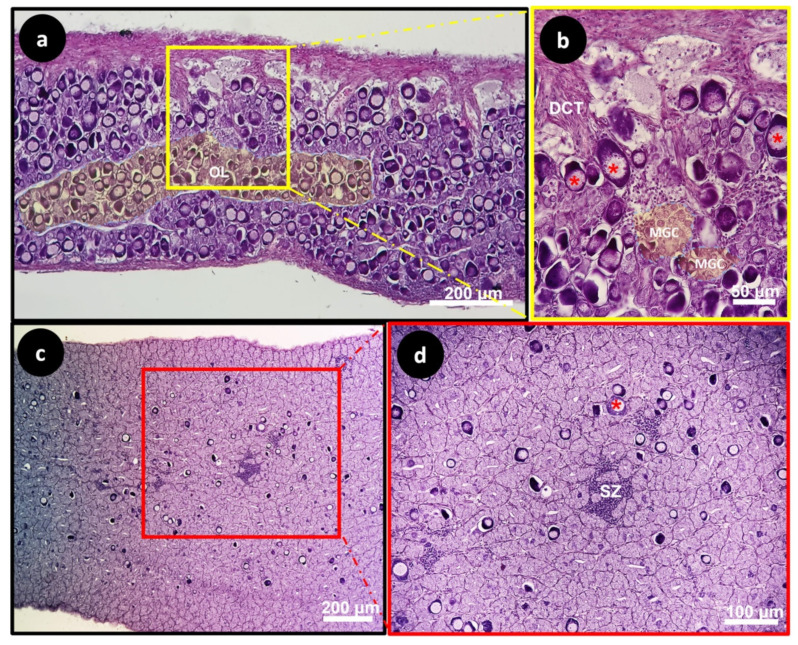
Light micrographs of European seabass gonads from control group CG: sagittal-medial section of ovary (**a**,**b**). The ovarian lamellae (OL) full of previtellogenic oocytes (Asterisk) and a few clusters of meiotic germinal cells (MGC), defining the edges of the ovarian cavity (white arrowhead), surrounded by dense connective tissues (DCT). Sagittal-medial section of testis (**c**,**d**) showing many intratesticular previtellogenic oocytes (Red Asterisk) and spermatozoon (SZ).

In both groups, the differentiated ovaries had well-developed ovarian lamellae, defining the edges of the ovarian cavity, and thus their definition as saculiform organs. The ovarian lamellae were full of previtellogenic oocytes containing wide nuclei (N) bordered by few nucleoli. (Figure 4a,b and Figure 5a,b). The 41.7% of EG subjects showed immature testes with type A spermatogonia located in the seminiferous lobules (Figure 4c,d). The remaining 60% of the CG fish showed testes with many intratesticular previtellogenic oocytes, dense connective tissue and spermatozoon (Figure 5c,d).

### 3.2. Immunohistochemical Results

**Figure 6:**
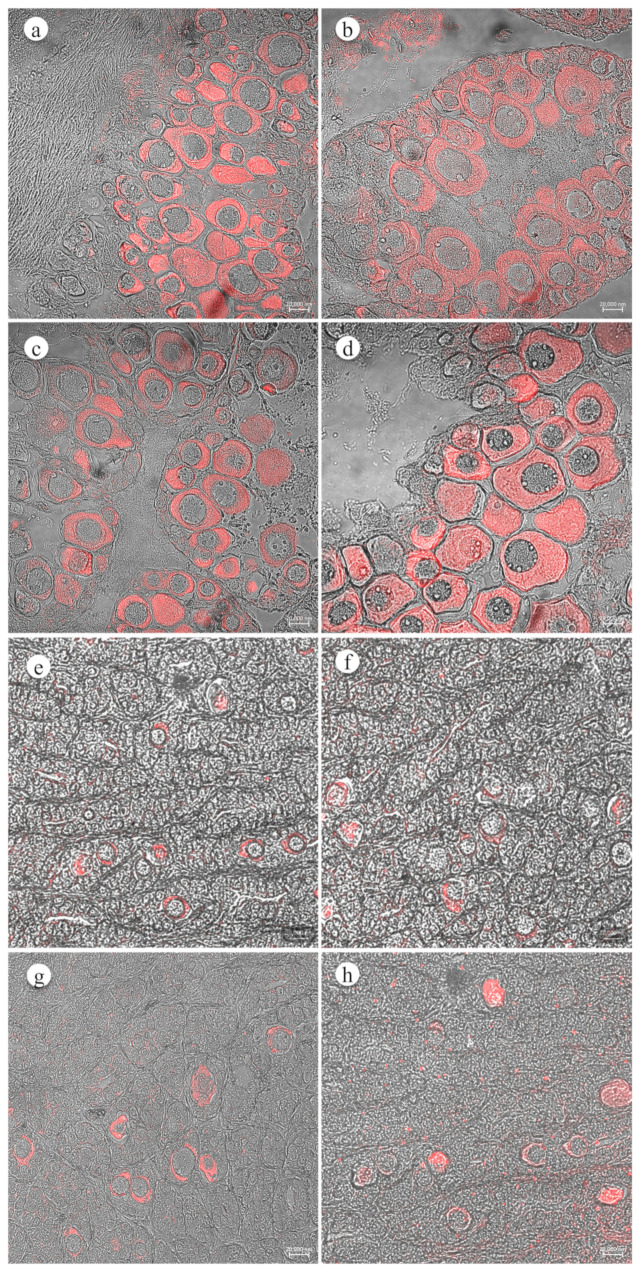
Immunohistochemical localization of ASIC2 and ASIC4 within European seabass ovaries and testes of CG and EG groups: (**a**) transmitted light and fluorescence photomicrograph of the ASIC2 immunolabeled previtellogenic oocytes from CG group; (**b**) transmitted light and fluorescence photomicrograph of the ASIC4 immunolabeled previtellogenic oocytes from EG group; (**c**) Transmitted light and fluorescence photomicrograph of the ASIC4 immunolabeled previtellogenic oocytes from CG group; (**d**) transmitted light and fluorescence photomicrograph of the ASIC4 immunolabeled previtellogenic oocytes from EG group; (**e**) transmitted light and fluorescence photomicrograph of the ASIC2 immunolabeled intratesticular previtellogenic oocytes from CG group; (**f**) transmitted light and fluorescence photomicrograph of the ASIC4 immunolabeled intratesticular previtellogenic oocytes from EG group; (**g**) transmitted light and fluorescence photomicrograph of the ASIC4 immunolabeled intratesticular previtellogenic oocytes from CG group; and (**h**) transmitted light and fluorescence photomicrograph of the ASIC4 immunolabeled intratesticular previtellogenic oocytes from EG group. Scale bars: 20,000 nm.

ASIC2 and ASIC4 were detected in European seabass gonads by indirect immunofluorescence (Figure 6). Control sections carried out, barring the primary antibody, did not reveal any immuno-labelling. The negative control for the immunofluorescence for both European seabass testis and ovary sections is shown in Figure S1. Both ASIC2 and ASIC4 were expressed in previtellogenic oocytes of ovaries and in scattered cells within some testes, most likely intratesticular previtellogenic oocytes in the CG and EG groups (Figure 6).

**Figure 7:**
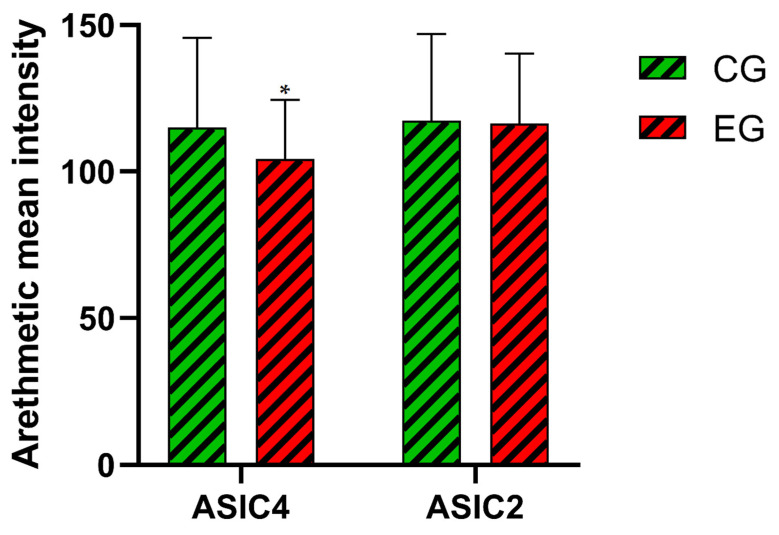
Arithmetic mean fluorescence intensity of Asic4 and Asic2 in European seabass CG and EG ovaries. Significances: * vs. CG (*p* < 0.05).

In an examination of ASIC2 and ASIC4 expression patterns, a quite discernible thermal influence was delineated (Figure 7, subjected to an ANOVA test with a significance threshold of *p* < 0.05). Indeed, fish challenged to a fast water-temperature increase (denoted as CG) manifested an augmented expression of ASIC4 relative to the EG cohort (*p* < 0.05). Moreover, it is imperative to underscore the equipoise observed in the arithmetic mean fluorescence intensities concerning ASIC2 between both the CG and EG cohorts.

## 4. Discussion

The development of sex in teleosts is plastic and prolonged. Interactions among environmental conditions, internal factors, and the genome itself may influence sex determination in fish species, suggesting that fish exhibit a polygenic system for sex determination [21]. Usually, following physiological conditions, genotype determines sex. Nevertheless, environmental factors affect phenotype and gonad differentiation during the entire lifecycle in some fish species.

The developmental rate of ontogeny in fish can be modified by progressive changes in water temperature during the larvae growing period [22], by body size, as well as by sexual differentiation [5,21], in some gonochoristic species, which are characterized by gonads with intersexual features [23].

The current study confirmed and reinforced the previously studied sex dimorphic growth pattern in European seabass [5,9,11]. Therefore, a significant sexual size dimorphism for females, in terms of body weight and standard length, was shown. Our results showed higher values for all biometric indices and a greater degree of differentiation in the female gonads in the EG than those in the CG. These findings reinforce the consolidated hypothesis within the scientific community that water-temperature manipulation during gonadal development influences sex differentiation and growth in seabass.

Data available in the literature showed controversial results regarding the sexual size dimorphism between sexes [9,11]. These controversial results could be related to the maintained temperatures in fish farming, as it has been established that sexual size dimorphism usually decreases under cold rearing temperatures in European seabass [24,25]. Indeed, the mechanism of fish gonadal sex differentiation is complex and regulated by multiple factors [26].

In the present study, an increase in the biometric indices analyzed was observed in seabass in the EG group compared to the CG group, and this phenomenon could be attributable precisely to the gradual increase in temperature during the experimental period in the experimental group, which probably stimulated growth as a result of the feed conversion rate and the enhanced metabolism [27]. A study focused on monitoring the individual growth of future subjects of both sexes starting from 23 mm standard length, to 83 dph, showed that differential growth in *D. labrax* can be observed from 96 to 103 dph (596 to 645 degree days above 10 °C, 27.5 to 30.3 mm standard lengths, 0.32 to 0.44 g body weight), and a possible sexual size dimorphism can be observed in support of the already existing female at 83 dph [28]. These recent findings support the hypothesis that female differentiation may be favored by a faster growth of the species due to the fact that sexual dimorphism in size seemed to occur before the first known indications of sexual differentiation. However, data available in the literature showed controversial results regarding the sexual size dimorphism between males and females [3,11,28,29,30].

In consideration of the correlation between sex and size, it is evident that larger fish species exhibit a prevalence of females whereas smaller ones tend to be predominantly male. This observation underscores the postulation that sex differentiation is more closely associated with length than age. The discernible influence of growth rate on sex differentiation further substantiates this relationship, irrespective of whether time and developmental thresholds have been crossed. It has previously been suggested that gonad maturation occurred after seabass reached between 9 to 12 cm in length [29]. This was in accordance with the histological analysis results of gonads collected from the four seabass specimens herein investigated. Indeed, all the analyzed fish showed gonads differentiated towards female or male lines. The histological analysis of gonads sampled from specimens belonging to EG showed that 58.3% of gonads differentiated towards the female line with differentiated ovaries with well-developed ovarian lamellae defining the edges of the ovarian cavity, full of previtellogenic oocytes. The remaining gonads sampled from this group showed sexually immature testes with type A spermatogonia observed in the seminiferous lobules.

The histological analysis of gonads sampled from specimens belonging to the CG group showed that 40% of gonads differentiated towards the female line with the ovarian lamellae full of previtellogenic oocytes and a few clusters of meiotic germinal cells defining the edges of the ovarian cavity. The fluxes of ions during oocyte maturation and fertilization are mediated by ion channels, transporters, and pumps. Most of them are localized at the plasma membrane; however, intra and intercellular channels are also fundamental players supporting cellular processes. Transport of Ca^2+^ through ion channels has been recognized as a critical step for oocyte maturation and egg activation [31].

Amiloride-sensitive sodium channels have been implicated in multiple reproductive and early developmental processes. These include the fast block of polyspermy in Xenopus oocytes and blastocoel expansion in mammalian embryo. In Drosophila, an Na+ selective channel known as dGNaC1 is specifically expressed in the gonads and early embryos, thus authors have postulated that it may be involved in oogenesis, spermatogenesis, and early embryonic development [18]. Moreover, Waldmann et al. [32] reported on the functional expression of a human isoform of an amiloride-sensitive Na+ channel in ovaries. The ASICs are members of the ENaC/degenerin family of the amiloride-sensitive Na+ channels superfamily, whose features include high permeability to Na that could be blocked by amiloride [17]. Many studies have reported that members of the ASIC gene superfamily could play a role in gametes differentiation and the post-fertilization development of eggs [18]. The current study reports on, for the first time, the expression of ASIC2 and ASIC4 in the previtellogenic follicles of European seabass ovaries. This finding highlights the importance of these ion channels on oocyte maturation and the early development of European seabass gonads. Indeed, ASICs could be implicated in the induction of temperature-dependent sex determination through a 17β-Estradiol (E2) temperature-induced differential expression. Another potential role for tASIC2 and ASIC4 in the previtellogenic follicles is their implication in promoting the proliferation and differentiation of the granulosa layer. In fact, this effect was reported in A549 lung cancer cells, where acidosis (pH < 7.0) led to increased cell migration, proliferation, and metabolic activity [20]. It has been suggested that the reported increase in cell proliferation and migration was due to the ASIC-activation-induced Ca^2+^ signaling [20]. Moreover, it has been shown that ASIC H(+)-gated are sensitive to temperature, which influences their kinetics [33]. Thus, the temperature sex determinism could be the result of Ca+ signaling induced by the temperature-modulated kinetics of ASICs channels.

The testes showed many intratesticular previtellogenic oocytes, dense connective tissue, and spermatozoon. Intratesticular oocytes were a common factor in many male subjects; however, their numbers did not increase but rather decreased after the completion of the testicular differentiation stage [2,3]. Intersexuality in the juvenile period is, to date, a poorly understood phenomenon. Gonads with intratesticular oocytes were not detected in the CG and this may be due to an advanced testicular differentiation phase. After this phase, growth of somatic tissue and an increase in spermatogonial density due to repeated mitosis were generally observed. As there are no longer ‘gonads’ that differentiate into oocytes, the density tends to progressively decrease until it becomes indistinguishable in the testicles. Apoptosis is also a phenomenon that can be traced back to the elimination of intratesticular oocytes [2]. The presence of gonads with intratesticular oocytes together with a lower percentage of ovaries in the CG than in the EG suggests that some species of intersex fish may become male and that intersexuality represents a manifestation of environmentally caused masculinization. Monitoring intersexuality may therefore prove useful for the interpretation of experimental results regarding sex determination in bass. Moreover, it has been demonstrated that the percentage of males exhibiting intratesticular oocytes was highly variable between families, suggesting that intersexuality might have a genetic basis in seabass [30].

The results available in the literature regarding the impact of rearing water temperatures on the ratio of males to females in seabass are controversial, probably because of the different thermosensitivity ranges in European seabass [29]. However, during the seabass’ exposure to low temperatures (13–15 °C) in the very early stages (30 h after fertilization) up to mid-metamorphosis, with the subsequent growth phase carried out at room temperature, a high percentage of females (70%) was observed [21]. Moreover, high temperatures during early life phases had a strong masculinizing effect in seabass, whereas low temperatures had a feminizing effect [21]. However, long-term low water temperature exposure during the period within which sexual differentiation takes place showed a masculinizing effect [30,34]. Moreover, if, during important phases such as fertilization, fish are subjected to low water temperatures, this can have a negative impact on muscle fiber development and thus on swimming performance. Exposure to low water temperatures immediately after fertilization can adversely affect muscle fiber development and thus swimming performance [35], while long-term exposure can also decrease growth [30,35], making both approaches difficult within the commercial aquaculture field. Water-temperature changes during early life in fish can lead to masculinization due to modulation of the expression of gene-encoding aromatase. Indeed, aromatase activity, which balances estrogens and androgens hormones, represent a switch for ovarian and testicular development in fish species, and the decrease in estrogen concentration and aromatase activity following treatment with this enzyme system inhibitor may lead to the reversal of the two sexes in Nile Tilapia [36,37] as well as in European seabass [38].

## 5. Conclusions

The results gathered in the current study strengthen the theory that water temperature can influence both gonadal differentiation and growth in the studied species. Moreover, the incidence of intratesticular oocytes highlighted intersexual cases strengthening the idea that sex differentiation in this species could be favored by changes in water temperature. Therefore, the water temperature index represents a leading external variable for the determination of male sexual intercourse observed in farmed seabass. Still, it is not particularly clear yet as to whether these phenomena have actual adaptive significance. The expression of acid-sensing ion channels (ASIC2 and ASIC4) reported for the first time in the ovaries of European seabass suggested their possible role in gonad differentiation and gamete development. Further studies on the genetic and endocrine factors as well as ASICs’ contribution, together with water temperature manipulation, on the sex determination of seabass are advocated. As a matter of fact, in-depth knowledge on the mechanism and factors around this phenomenon is crucial to better set up an increasingly innovative sea-bass-breeding protocol in order to increase the profitability of production.

## Figures and Tables

**Table 1:** Summary of the ASIC2 and ASIC4 identification and similarity searches.

Taxa	Max Score	Total Score	Query Cover	E Value	Per. Ident	Positives	Acc. Len	Accession
ASIC4								
*Homo sapiens*	-	-	-	-	-	-	539	Q96FT7-4
*Mus musculus*	109	109	100%	1.00 × 10^−33^	100.00%	100%	586	XP_036020902.1
*Rattus norvegicus*	108	108	100%	1.00 × 10^−33^	100.00%	100%	539	NP_071570.2
*Dicentrarchus labrax*	90.1	90.1	96%	7.00 × 10^−27^	75.51%	87%	505	A0A8P4FWP9
ASIC2								
*Homo sapiens*	-	-	-	-	-	-	512	Q16515-1
*Mus musculus*	72.4	72.4	97%	4.00 × 10^−21^	93.94%	100%	596	XP_006532059.1
*Rattus norvegicus*	73.2	73.2	97%	2.00 × 10^−21^	93.94%	100%	563	XP_032768433.1
*Dicentrarchus labrax*	53.9	53.9	100%	2.00 × 10^−14^	67.65%	85%	558	XP_051244300.1

**Table 2:** Mean values ± standard deviation (SD) of biometric indices (body weight, total length, and standard length) recorded from seabass (*Dicentrarchus labrax*) specimens belonging to control (CG) and experimental (EG) groups with the related statistical significances and differences on sex ratio.

Groups	Body Weight (g)	Total Length (cm)	Standard Length (cm)	Percent Females	Sex Ratio Differences
CG	68.8 ± 17.6 ^a^	17.4 ± 1.4 ^a^	15.1 ± 1.3 ^a^	40.0%	Χ^2^ = 6.48
EG	133.2 ± 38.1	22.2 ± 2.0	18.3 ± 2.3	58.3%	*p* = 0.01

Significant differences between groups (*p* < 0.01): ^a^ vs. EG.

## Data Availability

Data are contained within the article and Supplementary Materials.

## Supplementary Materials

The following supporting information can be downloaded at: https://www.mdpi.com/article/10.3390/ani14071024/s1, Figure S1: The negative control carried out barring the primary antibody on ovary (a) and testis slides (b) of European seabass.
