# Supplementary material for: Potential Implications of Acid-Sensing Ion Channels ASIC2 and ASIC4 in Gonadal Differentiation of Dicentrarchus labrax Subjected to Water Temperature Increase during Gonadal Development
Source: Animals (Basel). 2024 Mar 27;14(7):1024. doi: 10.3390/ani14071024 (PMC11010900; doi:10.3390/ani14071024)
Supplement: Supplementary file 1 [file animals-14-01024-s001.zip › animals-2883671-supplementary.pdf]

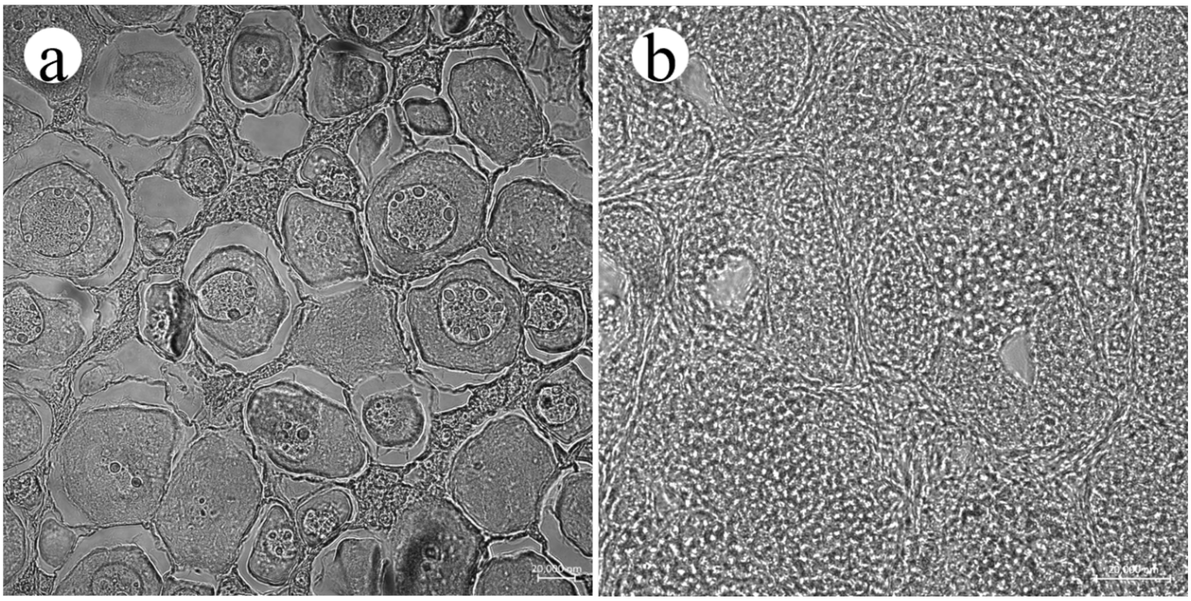

Figure S1: The negative control carried out barring the primary antibody on ovary (a) and testis slides (b) of European seabass.
